# A test of priority effect persistence in semi-natural grasslands through the removal of plant functional groups during community assembly

**DOI:** 10.1186/s12898-016-0077-9

**Published:** 2016-04-26

**Authors:** Kenny Helsen, Martin Hermy, Olivier Honnay

**Affiliations:** Plant Conservation and Population Biology, Department of Biology, University of Leuven, Arenbergpark 31, 3001 Heverlee, Belgium; Department of Biology, Norwegian University of Science and Technology, Høgskoleringen 5, 7034 Trondheim, Norway; Division Forest, Nature and Landscape Research, Department Earth and Environmental Sciences, University of Leuven, Celestijnenlaan 200E, 3001 Heverlee, Belgium

**Keywords:** Emergent groups, Functional traits, Graminoids, Historical contingency, Legumes, Niche modification, Niche preemption, Plant-soil feedback, Size-asymmetric competition, Soil legacies

## Abstract

**Background:**

It is known that during plant community assembly, the early colonizing species can affect the establishment, growth or reproductive success of later arriving species, often resulting in unpredictable assembly outcomes. These so called ‘priority effects’ have recently been hypothesized to work through niche-based processes, with early colonizing species either inhibiting the colonization of other species of the same niche through niche preemption, or affecting the colonization success of species of different niches through niche modification. With most work on priority effects performed in controlled, short-term mesocosm experiments, we have little insight in how niche preemption and niche modification processes interact to shape the community composition of natural vegetations. In this study, we used a functional trait approach to identify potential niche-based priority effects in restored semi-natural grasslands. More specifically, we imposed two treatments that strongly altered the community’s functional trait composition; removal of all graminoid species and removal of all legume species, and we compared progressing assembly with unaltered control plots.

**Results:**

Our results showed that niche preemption effects can be, to a limited extent, relieved by species removal. This relief was observed for competitive grasses and herbs, but not for smaller grassland species. Although competition effects acting within functional groups (niche preemption) occurred for graminoids, there were no such effects for legumes. The removal of legumes mainly affected functionally unrelated competitive species, likely through niche modification effects of nitrogen fixation. On the other hand, and contrary to our expectations, species removal was after 4 years almost completely compensated by recolonization of the same species set, suggesting that priority effects persist after species removal, possibly through soil legacy effects.

**Conclusions:**

Our results show that both niche modification and niche preemption priority effects can act together in shaping community composition in a natural grassland system. Although small changes in species composition occurred, the removal of specific functional groups was almost completely compensated by recolonization of the same species. This suggests that once certain species get established, it might prove difficult to neutralize their effect on assembly outcome, since their imposed priority effects might act long after their removal.

**Electronic supplementary material:**

The online version of this article (doi:10.1186/s12898-016-0077-9) contains supplementary material, which is available to authorized users.

## Background

Evidence continues to build that plant community assembly is rarely predictable at the species level, strongly challenging the traditional deterministic view of Clements [1] on succession [2, 3]. Indeed, many studies have shown that assembly outcome is not solely determined by abiotic conditions, but is partly unpredictable, often resulting in multiple alternative end states of the assembly process [4–6]. These observations support the view of stochastic community assembly, first discussed by Gleason [7] and Diamond [8], where assembly is expected to be, up to a certain extent, contingent upon historical processes [2, 9]. Historical contingency is hypothesized to affect assembly through multiple pathways, such as land-use legacies, interannual variation in (a)biotic conditions, historical landscape connectivity and priority effects [3, 10, 11]. In recent years, the importance of priority effects on plant assembly outcome has been given much attention [12–14]. Priority effects occur when early colonizing species inhibit or facilitate the establishment, growth or reproductive success of later arriving species, and are, for plants, hypothesized to be mainly caused either directly by size-asymmetric competition effects (inhibitory) [15, 16], or indirectly, by soil legacies (inhibitory or facilitative) [12, 17]. In the latter case the priority effects are effectuated by changes in nutrient availability, soil microbial communities or the buildup of allelochemicals [12, 16]. Soil legacies may persist for a long time after the causal species had disappeared from the community through the effects of plant-soil feedbacks [13, 17].

Even though priority effects are sometimes considered to be independent of species identity (neutral theory) [10, 18, 19], an increasing number of studies on both asymmetric competition effects and plant-soil feedbacks strongly suggests the opposite, with certain species seemingly strongly affected by priority effects exerted by specific species, while others remain unaffected by the same initial species [20–24]. It has, for this reason, been argued that the occurrence and severity of both inhibitory and facilitative priority effects are often strongly dependent upon a species niche. Fukami [14] more specifically hypothesizes that priority effects can be governed by two alternate niche-based processes, namely niche preemption (inhibitory) and niche modification (either inhibitory or facilitative). According to this framework, priority effects governed by niche preemption processes, such as size-asymmetric competition, will only affect species within niches, while niche modification based priority effects, such as soil legacies, will primarily act across niches [14]. Support for this niche preemption hypothesis has been found during experimental assembly of bacterial communities, where strong priority effects, and hence multiple community states, only emerged when species pools contained species with great niche overlap [25], and for nectar-inhabiting microorganisms, where priority effect size was significantly related to the extent of niche overlap [26]. Although direct evidence for this hypothesis is currently lacking for plant assembly, it has been experimentally shown that—at least certain—plant communities are most inhibitive to invasion of new species with niche requirements that are similar to those of species already present in those communities [27, 28]. Other studies, however, suggest that this limiting similarity process might not be universally applicable for plant communities [29].

Nonetheless, since functional plant traits are considered to be directly linked to a species niche, the functional group identity of species will likely greatly improve our predictive ability of priority effect presence and strength. Indeed, priority effects through size-asymmetric competition are expected to only occur within functional groups [26], while soil legacies likely affect species both within and among functional groups [14]. This potential predictive power of functional traits has already been illustrated by the observation of deterministic assembly at the functional trait (niche) level, as opposed to contingent assembly at the species identity level [30–32]. In these studies, the presence of multiple species with similar functional traits within a species pool are assumed to explain the occurrence of strong inhibitory priority effects at the species level through niche preemption, within each of the present niche spaces [33]. Nevertheless their potential, functional traits have only rarely been included as predictive variables in priority effect research during plant community assembly (but see [23, 34]).

The current knowledge on priority effects has been mainly gained through largely controlled mesocosm experiments looking either only at size-asymmetric competition (e.g., [10, 19, 24]) or at soil legacies (e.g., [22, 34, 35]). However, in natural systems, both inhibitory niche preemption and inhibitory or facilitative niche modification based priority effects may be simultaneously shaping community structures, making generalizations from these mesocosm experiments difficult [16]. Similarly, the priority effects observed in these mesocosm studies are usually surprisingly strong, likely because of optimal growing conditions and relatively short studied time scales (1–2 years after initial colonization) [10, 36]. Although little information is available on long-term priority effects, Hawkes et al. [22] have shown that experimental plant soil feedbacks can become increasingly negative for many species after 4 years. Two studies have furthermore observed indications of persistent priority effects at somewhat larger timescales (4–5 years) in grassland systems [32, 37]. A study of vernal pool plant communities in a more natural system, on the other hand, found the disappearance of priority effects after 7 years [20]. In conclusion, we can say that there is need for more in situ research to adequately quantify the importance of priority effects on long-term assembly progress and outcome in natural systems [19].

In this study, we want to fill part of this knowledge gap by evaluating potential priority effects at the functional trait level, using natural dry grasslands as a model system. More specifically, we evaluated small-scale plant community composition during the early stages of grassland development, following restoration practices. We imposed two treatments that severely altered the functional trait composition of the community; removal of all graminoid species and removal of all nitrogen fixating species (legumes). Additionally we also included a control treatment. The experiment was performed in four different restored semi-natural grasslands on the French-Belgian border, with four 5 × 5 m replicates (plots) of each treatment in each grassland, and was followed up during four consecutive years. In this experiment, we expected graminoids to mainly impose local inhibitory niche preemption (competition) effects on the community, since graminoids are often highly competitive. Legumes on the other hand, are known to fixate nitrogen, thus altering soil nutrient content. Taking into account the relatively weak competitive abilities of the legume species in our study system, we hypothesized that legumes mainly impose local facilitative niche modification (soil legacy) effects on the community. By comparing the changes in the species composition of both the treatment functional groups (graminoids and legumes) and previously defined functional trait groups (emergent groups) among the three treatments, we tried to verify the following hypotheses:

1. Removal of graminoids will relief local inhibitory within-niche competition effects, resulting in the local colonization of species with the same functional trait set as the removed species (inhibitory niche preemption).

2. Removal of legumes will likely result in the local colonization of species adapted to high nutrient availability, thus resulting in the colonization of species with a different functional trait set as the removed legume species (facilitative niche modification).

3. Small-scale plot level changes in the community composition caused by the graminoids and legumes will still be visible after 4 years, through newly enforced priority effects of secondary colonized species.

## Methods

### Study area

The study was performed in four recently restored semi-natural grassland patches, on the French-Belgian border (c. 50˚N, 4.5˚E). These patches are part of four larger, isolated grassland fragments, which are embedded in a matrix consisting of a mixture of arable land and forests, surrounded by several other grassland fragments. The four studied grassland patches were all restored from forest or shrub encroachment in 2007, and are adjacent to mature grassland within the grassland fragment. Initial restoration practices consisted of the complete removal of all aboveground vegetation and litter, after which spontaneous colonization of the bare soil was allowed. Soil characteristics were not directly altered, nor were plant species or seeds deliberately introduced to the restored sites. The follow up management of these grasslands consists of annual grazing by a migratory sheep flock. The grazing management prevents domination by woody species and also allows the dispersal of plant species through zoochory. Note that this setup strongly reduces any effect of dispersal limitation at the plot level, since all plots are imbedded within one of four larger grassland patches.

### Experimental design

To test for possible priority effects on grassland community assembly we used an experimental design consisting of three (functional identity) conditions. More specifically, these conditions consisted of the removal of all nitrogen fixation species or Fabaceae (*condition L; legumes*), removal of all Poaceaea, Cyperaceae and Juncaceae species (*condition G; graminoids*) and a standard condition with no manipulation of assembly (*condition C; control*). Manipulation for all conditions was performed within separate 5 × 5 m plots by carefully applying very small and targeted amounts of glyphosphate to the target species in the summer of 2010 (July), with a follow up in September of 2010 to remove the dead aboveground biomass and to make sure all treatment species were successfully killed. This set-up was replicated over four grassland patches. Each treatment was spatially randomly replicated for four times within each grassland patch, adding up to a total of 48, 5 × 5 m plots. After initial manipulation in 2010, these communities were allowed to follow spontaneous community assembly. Species composition and abundance (% cover) was collected for all plants (Tracheophytes) in each plot during the summer (July) of four consecutive years (2010–2013), with the 2010 data collected before initial manipulation. This field study was performed on public land. As our manipulations did not directly involve, nor did indirectly affect any endangered species we did not require special permission. The full plots × species dataset can be found at [38].

### Functional traits and emergent groups

For the evaluation of functional trait patterns, we used the emergent groups (EGs) that were defined by Helsen et al. [39] for the species pool of similar dry semi-natural grasslands. More precisely, Helsen et al. [39] delineated seven EGs based on twenty-eight functional plant traits using a minimum variance clustering method based on Gower’s similarity (Table 1). These traits were selected based on their relevance for community assembly, through their effects on species dispersal, establishment and persistence (cf. [40]) and were obtained from different databases (Additional file 1). Additionally, we calculated the community weighted means (CWM, as defined by [41]) for the three binary functional traits (thus the weighted proportions): nitrogen fixation, graminoid morphology and clonality. The values for these three functional traits were collected from the Ecoflora and Biolflor databases [42, 43].

**Table 1:** Overview of the emergent groups (EGs) as defined in Helsen et al. [39]

Emergent group	Group name	Characteristics
1	Megaphanerophytes	Long lived, shade-tolerant species, early flowering, wind pollinators, large seeds, transient seed bank, allogamous, anemo- and dysochores. Species of nutrient rich soils
2	Forest/shrub species	Long lived, shade-tolerant herbaceous and woody (understory) species, insect pollinated, transient seed bank, mixed mating system, few and heavy seeds, dysochores, large leaves. Species of nutrient rich soils, shade tolerant
3	Orchids	Many, small seeds, mycorrhiza-dependent
4	Small grassland herbs	Allogamous, shade intolerant, small herbs, autochores and zoochores, nitrogen fixators, semi-rosette species, specialists
5	Large herbs and grasses	Semi-rosette species, late flowering, large seeds, large species, large leaves, hemero- and zoochores, competitives. Species of nutrient rich soils
6	Sedges and shallow soil specialists	Mixed mating system, long seedbank longevity, small and light seeds, auto- and anemochores, mycorrhiza-independent
7	Annuals	Early flowering, autogamous, short-lived, small seeds and plants, zoochores, ruderals

For every group the name and typical functional trait values (characteristics) are given. Note that EG 3 (orchids) was not used in this study since too few species of this group were observed

### Diversity metrics

Species richness (S) and Pielou’s evenness index (E) were calculated for each plot, including all species, generalist species only, and specialist species only. Specialist species were defined as species confined to dry semi-natural grasslands in Belgium [44, 45] (Additional file 2). Species richness and total plant cover (%) were calculated twice for each EG separately, once including all species within each EG, and once excluding all treatment species (all Fabaceae, Poaceae, Cyperaceae and Juncaceae species).

### Species identity turnover

To evaluate changes in the species composition within treatment functional groups (graminoids and legumes), we quantified the species replacement of both species groups between the first (pre-treatment, year 0) and last year (year 3) of the experiment. Comparing species replacement of both species groups among the different treatments allows better insight in how priority effects are shaping the community composition. Species replacement was calculated as the ‘relativized species replacement’ R_rel_ based on presence-absence data [46] and as the ‘relativized abundance replacement’ ^a^R_rel_ based on abundance data [47] for both treatment functional groups separately, resulting in four species replacement measures.

### Statistical analysis

Differences in overall species diversity and species diversity of individual emergent groups between the three treatments were assessed using repeated measures linear mixed models (RMLMMs). More specifically, we constructed a separate RMLMM for each diversity metric as a dependent variable, including treatment, time (year) and the interaction between time and treatment as fixed factors, grassland identity as a random factor (‘variance components’ covariance type) and time (year) as a random repeated measure (‘unstructured’ covariance type). The model included both fixed and random (ID) intercepts and was based on restricted maximum likelihood (REML). Analogous RMLMMs were constructed for the CWM of nitrogen fixation, graminoid morphology and clonality. Prior to statistical analyses, several response variables were transformed to obtain normal distributions of the model residuals. In particular, all three measures of evenness (for all species, specialists and generalists) and the clonality CWM were squared, the cover of EG 1 was log transformed and we took the square root for the nitrogen fixation CWM, graminoid morphology CWM and the cover of all individual EGs (except EG 1) and total cover.

Among-treatment differences in species replacement within treatment functional groups were tested using linear mixed models (LMMs). More precisely, we constructed a separate LMM for each of the four calculated species replacement measures (R_rel_ and ^a^R_rel_) as a dependent variable, including treatment as a fixed factor and grassland identity as a random factor (‘variance components’ covariance type). The model included a fixed intercept and was based on restricted maximum likelihood (REML). Semi-partial R_β_^2^ coefficients were calculated for each covariate in all performed RMLMMs and LMMs, using the method of Edwards et al. [48].

## Results

**Fig. 1:**
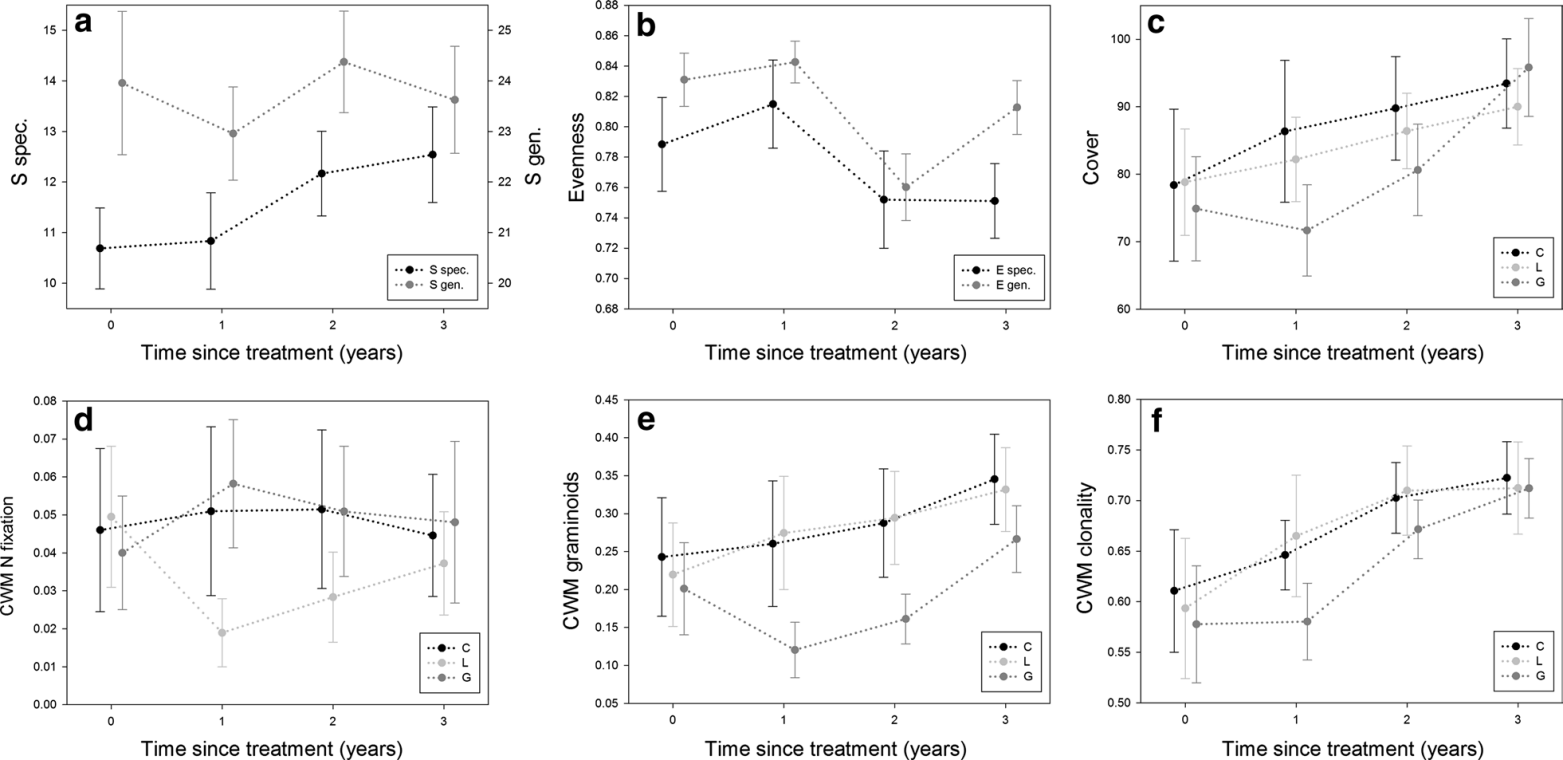
Change in species richness, evenness, total cover and CWM for several functional plant traits through time. Changes through time given for: **a** species richness, **b** Pielou’s evenness, **c** total cover, **d** CWM for nitrogen (N) fixation, **e** CWM for graminoids, **f** CWM for clonality. For **a** and **b** specialist species in *black*, generalist species in *grey*. For **c**–**f** *C* control *black*, *L* legumes *light grey*, *G* graminoids *dark grey*. Overall mean and 95 % confidence intervals are presented for each time point

Total species richness increased through time for all plots, independent of the treatment. This increase through time was found to be mainly driven by a strong increase in specialist species, with a decrease of generalist species (Table 2; Fig. 1a). The total evenness per plot, on the other hand, decreased through time, a pattern also observed for specialist species, and, although less pronounced, for generalist species (Fig. 1b). Evenness of specialist species was also affected by treatment, with a higher evenness in the G treatment (β G, Table 2). Total vegetation cover per plot strongly increased through time, but was reduced for the G treatment compared to the C treatment, in the first and second year following vegetation manipulation. At the third year following vegetation manipulation, no significant difference in total cover remained between the three treatments (significant interaction term, Table 2; Fig. 1c).

**Table 2:** Parameter estimates of the performed repeated measures linear mixed models on diversity measures and CWMs

	Time						Treatment					Interaction	
	F	R_β_^2^	β T0	β T1	β T2	β T3	F	R_β_^2^	β C	β L	β G	F	R_β_^2^
S	12.70***	0.22	34.63	33.13	35.82	36.13	0.15	<0.01	36.51	35.88	36.13	0.39	0.01
S spec.	18.74***	0.29	10.56	10.87	11.37	12.25	0.16	<0.01	12.81	12.56	12.25	1.38	0.03
S gen.	4.65**	0.09	24.07	22.25	24.44	23.88	0.01	<0.01	23.69	23.32	23.88	0.56	0.01
E^a^	28.16***	0.38	0.73	0.75	0.64	0.69	1.02	0.03	0.68	0.68	0.69	0.53	0.01
E spec.^a^	9.25***	0.17	0.73	0.79	0.68	0.62	13.67***	0.25	0.53	0.56	0.62	1.14	0.02
E gen.^a^	34.00***	0.43	0.69	0.70	0.57	0.65	1.70	0.05	0.69	0.66	0.65	0.54	0.01
Cover^b^	9.62***	0.18	13.62	13.33	14.15	15.44	1.43	0.04	15.25	14.97	15.44	3.42**	0.07
CWM N fix^b^	0.41	0.01	0.22	0.27	0.35	0.24	2.88°	0.08	0.24	0.22	0.24	8.26***	0.20
CWM graminoids^b^	35.80***	0.44	0.43	0.33	0.39	0.51	9.65***	0.21	0.58	0.57	0.51	12.86***	0.22
CWM clonality^a^	36.46***	0.45	0.35	0.34	0.45	0.51	1.87	0.04	0.53	0.52	0.51	2.47*	0.05

Beta-coefficient, test statistic and semi-partial R_β_^2^ given for time, treatment and the interaction term

*S* species richness, *E* Pielou’s evenness, *CWM* community weighted mean, *T* time since treatment (year), *C* control treatment, *L* legumes treatment, *G* graminoids treatment

° 0.10 ≥ *P* > 0.05 * 0.05 ≥ *P* > 0.01; ** 0.01 ≥ *P* > 0.001; *** 0.001 ≥ *P*

^a^Squared transformation

^b^Square root transformation

The CWM for nitrogen fixation did, overall, not change through time. However, when contrasting the three treatments, the CWM for nitrogen fixation decreased in the first year following vegetation manipulation in the L treatment, resulting in a significantly lower value for the L treatment compared to the C and G treatments in the first 2 years following vegetation manipulation. However, in the last year, no significant difference remained in the CWM for nitrogen fixation among the three treatments (Table 2; Fig. 1d). A comparable pattern was observed for the CWM of graminoid morphology, with an initial decrease for the G treatment compared to the C and L treatments in the first year following vegetation manipulation, and a gradual recovery of the CWM of graminoid morphology through time. Interestingly, unlike the CWM for nitrogen fixation, the CWM of graminoid morphology showed a gradual overall increase through time, independent of treatment (Table 2; Fig. 1e). The CWM for clonality showed the same patterns as the CWM for graminoid morphology, although the differences between the G treatment on the one hand and the C and L treatments on the other hand were less pronounced (Table 2; Fig. 1f).

An overview of the seven EGs defined by Helsen et al. [39] is presented in Table 1. Group names are based on the groups’ trait composition: megaphanerophytes (group 1), forest/shrub species (group 2), orchids (group 3), small grassland herbs (group 4), large herbs and grasses (group 5), sedges and shallow soil specialists (group 6) and annuals (group 7). Since too few species of EG 3 (orchids) were present in this study, it was removed from further analyses. Comparing the treatment functional groups (graminoids and legumes) with the EGs revealed that most legumes belong to EG 4 (small grassland herbs), and to lesser extent to EG 7 (annuals). Graminoids occur mainly in EGs 4 (small grassland herbs), 5 (large herbs and grasses) and 6 (sedges and shallow soil specialists), with also three species in EG 7 (annuals) (Additional file 2). Consequently, the RMLMMs for EG species richness and cover were only performed twice for these EGs (i.e., 4, 5, 6 and 7), with and without the treatment species (graminoids and legumes). For EGs 1 and 2, the RMLMMs were performed once for both species richness and cover (Table 3).

**Table 3:** Parameter estimates of the performed repeated measures linear mixed models for emergent groups species richness and cover

	Time						Treatment					Interaction	
	F	R_β_^2^	β T0	β T1	β T2	β T3	F	R_β_^2^	β C	β L	β G	F	R_β_^2^
S EG 1	9.16***	0.17	1.75	1.63	1.57	1.19	6.05**	0.13	1.19	1.63	1.19	0.93	0.02
S EG 2	3.93*	0.08	6.13	6.32	6.69	6.63	0.59	0.01	6.51	6.88	6.63	0.39	0.01
S EG 4	36.40***/28.02***	0.45/0.38	8.43/5.13	9.25/5.94	10.68/7.07	11.31/7.38	0.37/0.09	0.01/<0.01	12.12/7.63	12.06/7.88	11.31/7.38	0.26/0.18	0.01/<0.01
S EG 5	0.37/0.38	0.01/0.01	12.19/8.69	11.32/8.69	12.01/8.88	12.13/8.38	4.45*/3.72*	0.09/0.08	11.82/8.19	10.57/7.73	12.13/8.88	0.89/0.50	0.02/0.01
S EG 6	6.73***/8.03***	0.13/0.15	2.81/1.25	2.88/1.50	3.13/1.75	3.44/1.75	0.57/0.55	0.01/0.01	3.25/1.94	3.38/1.88	3.44/1.75	0.90/1.32	0.02/0.03
S EG 7	10.61***/7.93***	0.19/0.15	2.94/2.37	2.19/1.69	2.44/1.94	1.44/1.06	0.22/0.03	0.01/<0.01	1.69/1.25	1.63/1.44	1.44/1.06	1.14/1.14	0.02/0.02
cover EG 1^c^	9.69***	0.18	0.60	0.58	0.50	0.44	1.01	0.02	0.35	0.46	0.44	0.93	0.02
cover EG 2^b^	5.14**	0.10	6.87	6.62	6.27	6.37	0.37	0.01	5.71	5.76	6.37	0.41	0.01
cover EG 4^b^	24.19***/24.79***	0.35/0.36	5.81/4.59	6.78/5.69	8.27/7.06	9.31/7.69	0.11/0.10	<0.01/<0.01	8.64/7.07	8.63/7.30	9.31/7.68	2.02°/0.89	0.04/0.02
cover EG 5^b^	0.53/2.91*	0.01/0.06	8.20/6.62	7.49/6.86	7.58/6.88	8.07/6.57	0.44/2.18	0.01/0.05	8.15/6.00	7.71/5.67	8.07/6.57	2.71*/0.49	0.06/0.01
cover EG 6^b^	11.72***/5.36**	0.21/0.14	3.78/1.74	3.59/1.85	4.43/1.82	5.54/2.11	6.54**/0.48	0.14/0.01	6.52/2.25	6.28/2.16	5.54/2.11	2.31°/2.30°	0.05/0.07
cover EG 7^b^	3.53*/1.88	0.07/0.04	2.22/1.91	2.27/1.78	2.01/1.76	1.63/1.26	0.03/0.07	<0.01/<0.01	1.99/1.70	2.07/1.94	1.63/1.26	0.90/1.24	0.02/0.03

Beta-coefficient, test statistic and semi-partial R_β_^2^ given for time, treatment and the interaction term

For EG 4–7 analyses were performed first for all species in the respective EGs (results before slash) and second after excluding treatment species (graminoids and legumes) from the EGs (results after slash)

*S* species richness, *EG* emergent group (see Table 1 for EGs names and content), *T* time since treatment (year), *C* control treatment, *L* legumes treatment, *G* graminoids treatment

° 0.10 ≥ *P* > 0.05 * 0.05 ≥ *P* > 0.01; ** 0.01 ≥ *P* > 0.001; *** 0.001 ≥ *P*

^b^Square root transformation

^c^Logarithm transformation

**Fig. 2:**
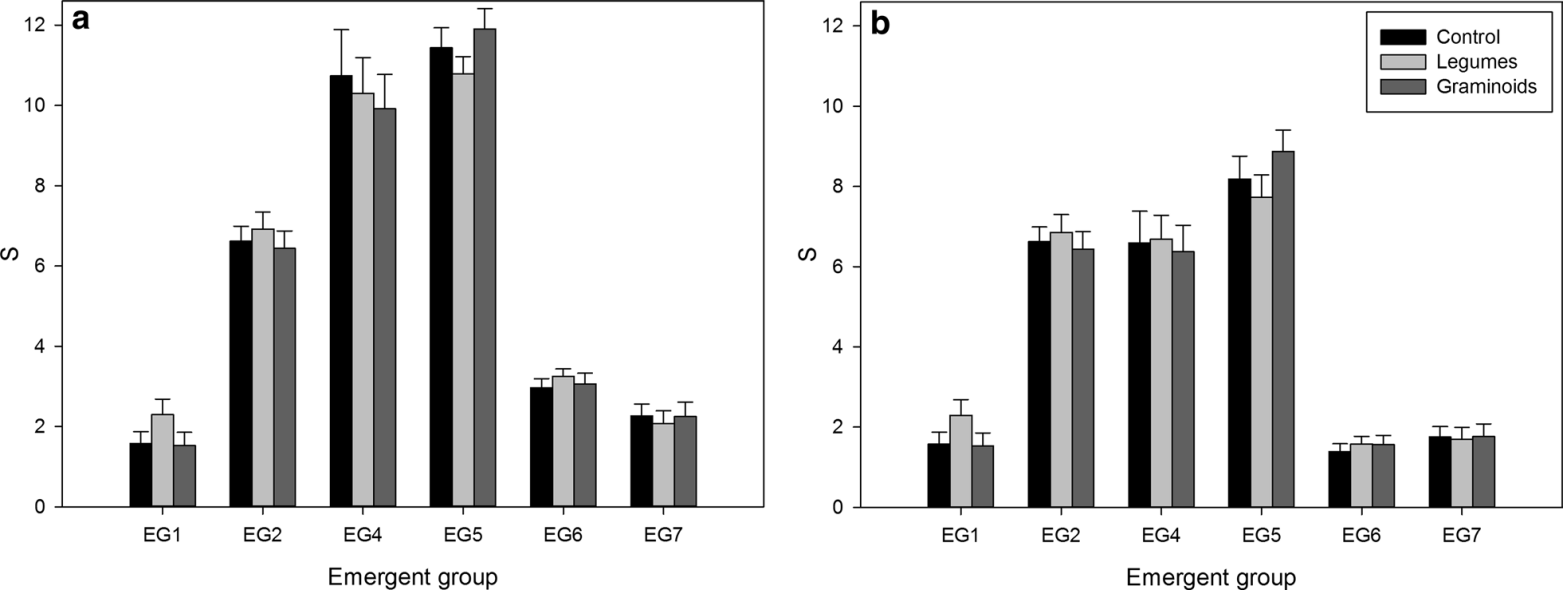
Differences in species richness for the different emergent groups between the three treatments. **a** *Bar plots* given for all present species, **b** *bar plots* given for all species excluding treatment species (graminoids and legumes). Differences are given for the different treatments separately (*C* control *black*, *L* legumes *light grey*, *G* graminoids *dark grey*). Overall mean and 95 % confidence intervals are presented for each emergent group. EG1 megaphanerophytes, EG2 forest/shrub species, EG4 small grassland herbs, EG5 large herbs and grasses, EG6 sedges and shallow soil specialists, EG7 annuals

Five of the six studied EGs were observed to change through time, both in terms of species richness and total cover, with a decrease of megaphanerophytes and annuals, and an increase in species richness of small grassland herbs and sedges and shallow soil specialists. The pattern for forest/shrub species was less pronounced, with a small increase in species richness, but a decrease in total cover through time. Large herbs and grasses were unaffected by time, both in terms of species richness and total cover (Table 3). Species richness of megaphanerophytes and large herbs and grasses furthermore differed among the treatments, with higher richness of megaphanerophytes, but lower richness of large herbs and grasses for the L treatment and higher richness of large herbs & grasses for the G treatment compared to the C treatment (Table 3; Fig. 2a). Most interestingly, these patterns remained significant after excluding the treatment species from the dataset (graminoids and legumes) (Table 3; Fig. 2b). Total cover of large herbs and grasses was lower for the G treatment compared to the C and L treatment in the second and third year following vegetation manipulation when including the treatment species (significant interaction term, post hoc results not shown). Total cover of sedges and shallow soil specialists was also significantly lower for the G treatment compared to the C and L treatment (significant treatment effect, Table 3). However, these differences disappeared when excluding the treatment species (Table 3).

Species replacement of graminoids, based on both species presence-absence and cover, during the first and last year of the experiment was not significantly different among treatments (Table 4). Similarly, species replacement of legumes between the first and last year of the experiment was similar among the three treatments (Table 4).

**Table 4:** Parameter estimates of the performed linear mixed models for species replacement

	Treatment				
	F	R_β_^2^	β C	β L	β G
G ^a^R_rel_ (cover)	0.12	0.003	0.36	0.40	0.38
G R_rel_ (presabs)	0.25	0.006	0.41	0.44	0.39
L ^a^R_rel_ (cover)	0.01	0.001	0.17	0.16	0.17
L R_rel_ (presabs)	0.25	0.006	0.17	0.13	0.17

Beta-coefficient, test statistic and semi-partial R_β_^2^ given for treatment

*G* graminoids, *L* legumes, ^*a*^ *R*_*rel*_ relativized abundance replacement, *R*_*rel*_ relativized species replacement, *C* control treatment, *presabs* presence–absence

* 0.05 ≥ *P* > 0.01; ** 0.01 ≥ *P* > 0.001; *** 0.001 ≥ *P*

## Discussion

### General assembly patterns

Changes in species richness and functional group composition through time observed in the C treatment can be interpreted as the natural assembly patterns in the studied grasslands. This natural assembly process is characterized by the replacement of generalist by specialist species and an increase in total vegetation cover through time. These results largely confirm the assembly patterns previously observed through a chronosequence approach in similar dry semi-natural grasslands [39]. At the functional trait level, the assembly patterns also partly confirmed the previous results of Helsen et al. [39]. However, opposite to the chronosequence study, the number of forest/shrub species showed a small increase, and annuals a strong decrease in species numbers through time in this study. Large herbs and grasses were found to remain relatively constant through time in this study, while a decrease in richness was found for these groups in the chronosequence study [39]. These differences might be caused by the smaller time scale in this study.

### Species removal effects on non-treatment species

The initial treatments resulted in changes in the functional trait set of the grassland communities in the first years following manipulation, with, for the G treatment, a reduction of total cover, graminoid species richness and associated cover of the emergent groups that mainly consist of graminoid species. In the L treatment, manipulation initially resulted in a reduction of N fixating species, but had no significant effect on total cover.

Interestingly, species richness was not affected by treatment, with similar levels of both specialist and generalist species across treatments. This is in accordance with other priority effect experiments, where species richness was found to converge, independent of initial differences in species richness and treatment [10, 32]. Both the G and L treatments nevertheless affected species composition. More specifically, the removal of graminoids resulted in a small, but nonetheless significant increase in the number of species in EG 5 (large herbs and grasses). This is in accordance with our (inhibitory) niche preemption hypothesis, with species with similar niches as the removed graminoids benefitting from the treatment [26, 28]. In other words, the removal of large competitive grasses resulted in a small increase of large competitive herbs, likely through colonization. The absence of a similar pattern for EGs 4 and 6, which also contain many graminoids, is likely caused by the fast recovery of graminoids species in these grasslands, well before other species can colonize due to reduced within-niche competition (see the ‘species removal effects on treatment species’ discussion section further). Alternatively, it could be argued that the inhibitory competitive priority effects are less pronounced within these EGs, which are characteristic of high stress-low competition communities. It has indeed been suggested that the strength of direct (inhibitory niche preemption) priority effects are dependent on soil nutrient levels, implying that priority effects in experimental studies (using optimal nutrient concentrations) are likely much stronger than those occurring in natural (nutrient poor) communities [10, 36]. Since EG 5 mainly consists of relatively competitive species, this could explain the stronger effect of species removal for this specific group. Indeed, all else being equal, direct inhibitory niche preemption priority effects are expected to be more pronounced for competitive species that produce much biomass [24, 36]. Since soil legacies are strongly species-specific, we also cannot exclude the possibility of differential facilitative or inhibitory soil legacy effects of graminoid species, indirectly promoting the establishment of species of EG 5 [21, 22].

In accordance with our hypothesis, legume removal did not affect the species richness of EG 4 (or 7), which contain all legume species, but resulted in changes in unrelated functional groups (decrease of the number of large herbs and grasses, and an increase of megaphanerophytes). This suggests the occurrence of niche modification effects of legumes after their removal. As discussed earlier, the low overall competitive abilities of the legumes present in these grasslands (Additional file 2) likely explain why competitive exclusion (niche preemption) is limited within this functional group. Previous research has also shown that legumes do often not exert persistent inhibitory priority effects through size-asymmetric competition, and often facilitate higher biomass production of functionally different co-occurring plant species through nitrogen enrichment of the soil (facilitation) [19, 49]. This facilitative niche modification effect can be especially effective for plants growing in nutrient poor grasslands, as is the case in this study. The removal of legumes in the L treatment likely resulted in open patches with increased nitrogen availability. Megaphanoreophyte seedlings seem to be better at establishing at these former legume sites, suggesting facilitative soil legacies through nitrogen enrichment [21]. In this scenario, the observed decrease in species of EG 5 might be partly caused by the decreased competitive success of large (herbs and grasses) against megaphanerophytes. Alternatively, the absence of legumes might have resulted in a lower overall availability of nitrogen in the community, an effect that will most strongly inhibit the growth of species that are not adapted to nutrient poor conditions, such as those of EG 5. Indeed, this EG mainly contains generalist species adapted to fast growth and relatively nutrient rich soils (Table 1; [39]). The positive effect on megaphanerophytes on the other hand might then suggest that legumes have a negative effect on tree and shrub seedlings through inhibitory niche modification effects, independent of their effect on nitrogen availability.

### Species removal effects on treatment species

In this experiment, both graminoid and legume removal was after 4 years almost completely compensated by the recolonization of graminoids and legumes, respectively, strongly suggesting that niche processes shape community assembly and priority effects in certain semi-natural grassland systems, as previously argued by Helsen et al. [31]. Although this was largely expected for graminoids, we did not expect similar patterns to occur for legumes. More surprisingly, the species replacement rates among the treatments show that this recolonization is effectuated by largely the same set of species as those that were removed. This suggests that niche preemption through size-asymmetric competition is likely only partly driving these patterns, since we would have expected some levels of species replacement (within functional groups) in this case. Likely, the observed patterns are also partly driven by localized soil legacies that promote the colonization of the same species (facilitative), or prevent the colonization of other species (inhibitory legacy effects acting within a functional group). Although some studies demonstrated that within species plant-soil feedbacks can be inhibitory [21, 35], other studies have indeed shown that many species exhibit weaker inhibitory, or even facilitative plant-soil feedbacks upon conspecifics compared to plant-soil feedbacks upon other species [23, 34].

The observed patterns can, however, also be at least partly explained by other confounding factors. Since (dead) belowground biomass of the treatment species was not actively removed, possible priority effects of these species might have been much stronger than would have been the case after complete removal of the species. Indeed, inhibitory size-asymmetric competitive priority effects are not solely driven by aboveground biomass, but can also remain strong when aboveground biomass is periodically removed through mowing [19]. Furthermore, since all treatments were performed in relatively small plots within a larger grassland, the removed species are also present in the direct vicinity of the treatment plot, enhancing the chances of recolonization of the plot by the same species set, thus deflating replacement rates. This effect might have been especially strong for legumes, since only a relatively small number of species was present in these grasslands. Most of the graminoids were furthermore strongly clonal (Fig. 1e, f), also allowing quick clonal recolonization of the plot by ramets present at the vicinity of the plot border. In conclusion, we believe that soil legacies likely resulted in reduced levels of species replacement, but that this affect was likely not as strong as suggest by the species replacement results.

### Treatment effects through time

Although changes in the EG compositions across the different treatments persisted after 4 years (no significant interaction between time and treatment), we did also observe a fast recovery of the number and composition of both legumes and graminoids during the same time span. Contrary to our predictions, this suggests that the effect of specific functional group removal during grassland assembly does not result in alternative assembly pathways, through newly enforced priority effects of the secondary colonized species. These results more likely suggest that soil legacies result in, at least partial, maintenance of initial priority effects after species removal. This, in turn, allows the fast recolonization of the removed species, with only limited changes in overall species composition. These results are partly in agreement with the study of Plückers et al. [37], where initial differences in species richness and functional composition (forbs, grasses and legumes) through differential seeding, became very small after 4 years, with communities seemingly converging toward similar species richness and functional composition.

## Conclusions

In this study, we explored how priority effects within and among functional groups affect community assembly during natural plant community assembly. More specifically, our results show that, in a low nutrient, (semi-)natural grassland system, inhibitory priority effects acting through niche preemption can be slightly relieved by species removal. However, this relief depended on the competitive ability of the removed species, with relief only observed for more competitive grasses and herbs, but not for smaller grassland specialists. Although competition effects acting within functional groups (niche preemption) were observed for graminoids, they do not seem to apply to legumes. Indeed, the removal of legumes mainly affected functionally unrelated generalist species and megaphanerophytes, likely through the facilitative niche modification effects of nitrogen fixation after legume removal [14].

On the other hand, species removal was, contrary to our expectations, almost completely compensated by recolonization of the same species set, suggesting that the net community composition effects of species (group) removal is rather limited in this natural system. This additionally suggests that soil legacies are, at least up to a certain extent, important drivers of assembly patterns during natural grassland assembly. We can expect that, in the context of ecological restoration, if unwanted species get established, it might prove difficult to neutralize their effect on the community assembly outcome, since their imposed priority effects might act long after their removal through imposed soil legacies [16].

## Additional files

10.1186/s12898-016-0077-9 Overview of the selected traits, used for the delineation of the emergent groups. Description, scale and main data sources are given for every trait.
10.1186/s12898-016-0077-9 Overview of the species list. Species are defined as generalist (g) of specialist (s), and subdivided in seven emergent groups (EGs). Group numbers correspond to the emergent groups described in Table 1. The treatment column indicates what species are graminoids (G) and legumes (L).

## Abbreviations

^a^R_rel_: relativized abundance replacement
C: control treatment
CWM: community weighted mean
E: Pielou’s evenness
EG: emergent group
G: graminoids treatment
L: legumes treatment
LMM: linear mixed model
REML: restricted maximum likelihood
RMLMM: repeated measures linear mixed model
R_rel_: relativized species replacement
S: species richness
