# Supplementary material for: A test of priority effect persistence in semi-natural grasslands through the removal of plant functional groups during community assembly
Source: BMC Ecol. 2016 Apr 26;16:22. doi: 10.1186/s12898-016-0077-9 (PMC4847182; doi:10.1186/s12898-016-0077-9)
Supplement: Supplementary file 1 — 10.1186/s12898-016-0077-9 Overview of the selected traits, used for the delineation of the emergent groups. Description, scale and main data sources are given for every trait. [file 12898_2016_77_MOESM1_ESM.pdf]

Additional file 1. Overview of the selected traits, used for the delineation of the emergent groups. Description, scale and main data sources are given for every trait.

Trait	Description	Scale	Data source
plant height	mean individual height (m)	ratio	Lambinon *et al*. 1998
life span	1. annual; 2. biennial; 3. once flowering perennial; 4. multiple flowering perennial	nominal	Klotz *et al*. 2002
rosette type	1. rosette; 2. half-rosette; 3. no rosette	nominal	Klotz *et al*. 2002
L	Ellenberg light value: ranging from 1 (plants of deep shade) to 9 (plants of full sun)	ordinal	Kleyer *et al*. 2008
N	Ellenberg nutrient value: ranging from 1 (plants of nutrient poor soils) to 9 (plants of nutrient rich soils)	ordinal	Kleyer *et al*. 2008
flowering start	Month in which flowering begins	ordinal	Klotz *et al*. 2002
pollen vector	1. insects; 2. selfing; 3. wind; 4. water	nominal	Klotz *et al*. 2002
reproductive type	1. mainly seeds/spores; 2. seeds/spores & vegetative; 3. mainly vegetative	nominal	Klotz *et al*. 2002
diaspore type	1. fruit; 2.seed; 3. spore; 4. vegetative	nominal	Klotz *et al*. 2002
seed length	length of a single seed	ratio	Kleyer *et al*. 2008
seed shape	seed length/width ratio	ratio	Kleyer *et al*. 2008
seed longevity	short and long term persistent seed records proportional to the total number of records per species	ratio	Thompson *et al*. 1997, Bekker *et al*. 1998
fertility system	1. autogamous; 2. mixed mating system; 3. Allogamous	nominal	Klotz *et al*. 2002
seed mass	mass of a single seed (mg)	ratio	Kleyer *et al*. 2008
seed number	logarithmic transformed number of seeds per plant	ratio	Kleyer *et al*. 2008
mycorrhizal frequency	0. never; 1. rarely; 2. occasionally; 3. normally	ordinal	Fitter & Peat 1994
mycorrhizal type	1. arbuscular; 2. ecto; 3. orchid	nominal	Fitter & Peat 1994
autochory	0. no autochory; 1. Self dispersal	binary	Kleyer *et al*. 2008
hemerochory	0. no hemerochory; 1. Seed dispersal by human action	binary	Kleyer *et al*. 2008
anemochory	0. no anemochory; 1. Seed dispersal by wind	binary	Kleyer *et al*. 2008
endozoochory	0. no endozoochory; 1. Seed dispersal after digestion	binary	Kleyer *et al*. 2008
epizoochory	0. no epizoochory; 1. Adhesive dispersal by animals	binary	Kleyer *et al*. 2008
dysochory	0. no dysochory; 1. Seed dispersal by scatter-hoarding animals	binary	Kleyer *et al*. 2008
nitrogen fixation	1. nitrogen fixators; 2. non fixators	binary	Poschlod *et al*. 2003
age first flowering	1. < 1 year; 2. between 1 and 5 years; 3. > 5 years	ordinal	Kleyer *et al*. 2008
leaf size	mean leaf size (mm2)	ratio	Kleyer *et al*. 2008
A.P. sheep	Seed attachment potential to sheep wool (%)	ratio	Römermann *et al*. 2005, Cappers *et al*. 2006
A.P. cattle	Seed attachment potential to cattle hair (%)	ratio	Römermann *et al*. 2005, Cappers *et al*. 2006

References Additional file 1.

Bekker RM, Bakker JP, Grandin U, Kalamees R, Milberg P, Poschlod P, et al. Seed size, shape and vertical distribution in the soil: indicators of seed longevity. *Funct Ecol.* 1998; **12**:834–842.

Cappers RTJ, Bekker RM, Jans JEA. Digitale zadenatlas van Nederland. Groningen: *Groningen Archaeological Studies 4, Barkhuis Publishing*, www.zadenatlas.nl; 2006.

Fitter AH, Peat HJ. The ecological flora database. *J Ecol.* 1994; **82**:415–442.

Kleyer M, Bekker R, Knevel IC, Bakker JP, Thompson K, Sonnenschein M, et al. The LEDA Traitbase: a database of life-history traits of the Northwest European flora. *J Ecol.* 2008; **96**:1266–1274.

Klotz S, Kühn I, Durka W. BIOLFLOR - Eine Datenbank zu biologisch-ökologischen Merkmalen der Gefäßpflanzen in Deutschland. Bonn: Bundesamt für Naturschutz, Schriftenreihe für Vegetationskunde 38; 2002.

Lambinon J, De Langhe J, Delvosalle L, Duvigneaud J. Flora van België, het Groothertogdom Luxemburg, Noord-Frankrijk en de aangrenzende gebieden (Pteridofyten en Spermatofyten). Meise: Nationale plantentuin van België; 1998.

Poschlod P, Kleyer M, Jackel A-K, Dannemann A, Tackenberg O. BIOPOP — A database of plant traits and internet application for nature conservation. *Folia Geobot.* 2003; **38**:263–271.

Römermann C, Tackenberg O, Poschlod P. How to predict attachment potential of seeds to sheep and cattle coat from simple morphological seed traits. *Oikos.* 2005; **110**:219–230.

Thompson K, Bakker JP, Bekker RM. The soil seed banks of north west Europe: methodology, density and longevity. Cambridge: Cambridge University Press; 1997.
