# Supplementary material for: A test of priority effect persistence in semi-natural grasslands through the removal of plant functional groups during community assembly
Source: BMC Ecol. 2016 Apr 26;16:22. doi: 10.1186/s12898-016-0077-9 (PMC4847182; doi:10.1186/s12898-016-0077-9)
Supplement: Supplementary file 2 — 10.1186/s12898-016-0077-9 Overview of the species list. Species are defined as generalist (g) of specialist (s), and subdivided in seven emergent groups (EGs). Group numbers correspond to the emergent groups described in Table 1. The treatment column indicates what species are graminoids (G) and legumes (L). [file 12898_2016_77_MOESM2_ESM.pdf]

Additional file 2. Overview of the species list. Species are defined as generalist (g) of specialist (s), and subdivided in 7 emergent groups (EGs). Group numbers correspond to the emergent groups described in Table 1. The treatment column indicates what species are graminoids (G) and legumes (L).

species	spec/gen	EG	treatment
Acer campestre	g	1	
Acer pseudoplatanus	g	1	
Agrimonia eupatoria	s	2	
Agrostis capillaris	g	5	G
Agrostis stolonifera	g	5	G
Anagallis arvensis	g	7	
Anthyllis vulneraria	s	4	L
Arabis hirsuta	s	6	
Arctium sp.	g	7	
Arenaria serpyllifolia	s	7	
Arrhenatherum elatius	g	4	G
Arum maculatum	g	2	
Atropa bella-donna	s	2	
Betula pendula	g	1	
Betula pubescens	g	1	
Brachypodium pinnatum	s	5	G
Brachypodium sylvaticum	g	5	G
Briza media	s	4	G
Bromus erectus	s	5	G
Bromus hordeaceus	g	5	G
Bromus sterilis	g	5	G
Buxus sempervirens	s	2	
Calystegia sepium	g	5	
Campanula rotundifolia	g	4	
Cardamine hirsuta	g	7	
Carduus crispus	g	5	
Carex caryophyllea	s	6	G
Carex digitata	s	6	G
Carex divulsa	g	6	G
Carex flacca	s	6	G
Carex hirta	g	6	G
Carex humilis	s	6	G
Carex pilulifera	g	6	G
Carex sylvatica	g	6	G
Carpinus betulus	g	1	
Catapodium rigidum	s	7	G
Centaurea scabiosa	s	4	
Centaurium erythraea	g	5	
Cerastium fontanum	g	6	
Chenopodium sp.	g	7	

Cirsium acaule	s	4	
Cirsium arvense	g	5	
Cirsium palustre	g	5	
Cirsium vulgare	g	5	
Clematis vitalba	s	5	
Clinopodium acinos	s	4	
Clinopodium vulgare	s	4	
Convolvulus arvensis	g	4	
Conyza canadensis	g	5	
Cornus sanguinea	g	2	
Corylus avellana	g	1	
Cotoneaster horizontalis	g	2	
Crataegus monogyna	g	2	
Crepis biennis	g	5	
Crepis capillaris	g	5	
Dactylis glomerata	g	4	G
Danthonia decumbens	g	4	G
Daucus carota	g	5	
Digitalis lutea	s	6	
Dipsacus fullonum	g	5	
Dryopteris carthusiana	g	2	
Dryopteris filix-mas	g	2	
Echium vulgare	s	4	
Epilobium montanum	g	5	
Epilobium parviflorum	g	5	
Epilobium tetragonum	g	7	
Euonymus europaeus	g	2	
Eupatorium cannabinum	g	5	
Euphorbia cyparissias	s	4	
Festuca lemanii	s	4	G
Fragaria vesca	g	4	
Fraxinus excelsior	g	1	
Galeopsis tetrahit	g	7	
Galium aparine	g	7	
Galium mollugo	g	4	
Galium pumilum	s	4	
Genista sagittalis	s	4	L
Geranium columbinum	s	7	
Geranium dissectum	g	7	
Geranium robertianum	g	7	
Geum urbanum	g	5	
Hedera helix	g	1	
Helianthemum nummularium	s	4	
Helictotrichon pubescens	g	5	G
Helleborus foetidus	s	2	
Heracleum sphondylium	g	2	

Hieracium murorum	g	5	
Hieracium pilosella	g	5	
Hippocrepis comosa	s	4	L
Holcus lanatus	g	5	G
Holcus mollis	g	5	G
Hordeum murinum	g	7	G
Hypericum perforatum	g	6	
Hypochaeris radicata	g	4	
Inula conyzae	s	5	
Jacobaea vulgaris	g	5	
Juglans regia	g	1	
Knautia arvensis	g	4	
Koeleria macrantha	s	5	G
Lactuca muralis	g	7	
Lactuca serriola	g	5	
Lapsana communis	g	7	
Leontodon autumnalis	g	5	
Leontodon hispidus	s	5	
Leucanthemum vulgare	g	4	
Ligustrum vulgare	g	2	
Linum catharticum	s	7	
Lolium perenne	g	5	G
Lonicera periclymenum	g	2	
Lotus corniculatus	g	4	L
Malva moschata	s	6	
Medicago lupulina	g	4	L
Melica ciliata	s	4	G
Melilotus altissimus	g	4	L
Mercurialis annua	g	7	
Myosotis arvensis	g	7	
Origanum vulgare	s	4	
Ornithogalum umbellatum	g	2	
Papaver rhoeas	g	7	
Phleum bertolonii	s	5	G
Phleum pratense	g	5	G
Picris hieracioides	s	5	
Pimpinella saxifraga	g	4	
Pinus sylvestris	g	1	
Plantago lanceolata	g	4	
Plantago major	s	5	
Plantago media	s	4	
Platanthera chlorantha	s	3	
Poa angustifolia	g	5	G
Poa annua	s	7	G
Poa compressa	g	5	G
Poa pratensis	s	5	G

Poa trivialis	g	5	G
Polygala comosa	s	4	
Polygala vulgaris	s	4	
Polygonatum odoratum	g	2	
Polygonum aviculare	g	7	
Populus tremula	s	3	
Potentilla neumanniana	s	4	
Potentilla reptans	g	4	
Primula veris	s	4	
Prunella vulgaris	g	4	
Prunus avium	g	1	
Prunus spinosa	g	2	
Quercus robur	g	1	
Ranunculus acris	g	4	
Ranunculus repens	s	4	
Reseda luteola	g	7	
Rhamnus cathartica	g	2	
Rosa canina	s	2	
Rosa rubiginosa	g	2	
Rubus fruticosus	g	2	
Rubus idaeus	g	2	
Rumex crispus	g	5	
Rumex obtusifolius	g	5	
Salix caprea	g	3	
Salix cinerea	g	3	
Sanguisorba minor	s	4	
Scabiosa columbaria	s	4	
Sedum acre	s	6	
Sedum album	s	6	
Senecio erucifolius	g	5	
Senecio vulgaris	g	7	
Sesleria caerulea	g	4	G
Solanum dulcamara	g	2	
Solanum nigrum	g	2	
Sonchus asper	g	7	
Sonchus oleraceus	s	5	
Stachys alpina	s	5	
Stachys officinalis	g	4	
Tanacetum vulgare	g	5	
Taraxacum officinalis	g	5	
Teucrium chamaedrys	s	4	
Teucrium montanum	s	4	
Teucrium scorodonia	g	2	
Thlaspi perfoliatum	s	7	
Thymus pulegioides	g	4	
Tilia platyphyllos	g	1	

Torilis japonica	g	2	
Tragopogon pratensis	g	5	
Trifolium campestre	g	7	L
Trifolium pratense	g	4	L
Trifolium repens	g	4	L
Trisetum flavescens	s	6	G
Tussilago farfara	g	5	
Urtica dioica	g	5	
Valeriana officinalis	g	5	
Verbascum lychnitis	g	5	
Verbascum nigrum	g	5	
Verbascum thapsus	g	5	
Veronica arvensis	g	7	
Veronica officinalis	s	4	
Veronica prostrata	s	4	
Viburnum lantana	g	2	
Vicia cracca	g	4	L
Vicia hirsuta	g	7	L
Vicia sativa	g	7	L
Vincetoxicum hirundinaria	s	2	
Viola hirta	g	2	
Viola riviniana	g	2	
Vulpia myuros	g	4	G
